# Complete Genome Sequence of Campylobacter fetus subsp. testudinum Strain 772, Isolated from Ascites of a Patient with Chronic Kidney Disease

**DOI:** 10.1128/genomeA.00432-18

**Published:** 2018-05-31

**Authors:** Shui-ping Hou, Peng He, Yong Zhou, Xin-wei Wu

**Affiliations:** a Department of Microbiology, Guangzhou Center for Disease Control and Prevention, Guangzhou, China

## Abstract

Campylobacter fetus subsp. testudinum originating in reptiles can cause invasive infections in humans. Here, we present the whole-genome sequence of C. fetus subsp. testudinum strain 772, isolated from a human patient in China.

## GENOME ANNOUNCEMENT

Campylobacter fetus is a Gram-negative slender spiral bacterial pathogen that can cause abortion or infertility in animals and extraintestinal infections (bacteremia, meningitis, arthritis, etc.) in humans (1). Based on traditional biochemical and genotyping methods, Campylobacter fetus is currently divided into three subspecies: C. fetus subsp. fetus and C. fetus subsp. venerealis, associated with infections in mammals, and C. fetus subsp. testudinum, primarily isolated from reptiles (2, 3). Previous studies demonstrated genetic diversities among the three subspecies and that the genetic divergence between mammal- and reptile-associated C. fetus is more extensive than that within mammal-associated C. fetus (4, 5). Here, we report the whole-genome sequence of C. fetus subsp. testudinum strain 772, which was isolated from ascites of a patient with chronic kidney disease, to further characterize the genomics of the subspecies.

Strain 772 was enriched in Bolton broth by incubating microaerobically at 37°C for 72 h. Genomic DNA was extracted using the QIAamp DNA minikit (Qiagen, Germany), and the purity was evaluated using a Qubit 3.0 fluorometer (Thermo, USA). The TruSeq DNA sample prep kit (Illumina) was used to construct the libraries for genome sequencing. Sequencing was performed on an Illumina HiSeq platform using the TruSeq PE cluster kit version 3 and TruSeq SBS kit. A total of 20,490,024 reads were generated, resulting in 1,649× sequencing coverage. The genome sequence was assembled using the Celera Assembler 8.0. After the first round, GapCloser (version 1.12) was used for gap closing, and all the contigs were concatenated into a single scaffold of a genome sequence.

The genome of C. fetus subsp. testudinum strain 772 consists of 1,863,540 bp, with a mean G+C content of 33.13%. No plasmids were identified. The open reading frames (ORFs) were predicted using Glimmer version 3.02. The rRNA and tRNA genes were identified using the RNAmmer1.2 and tRNAscan-SE 1.23 software, respectively. The genome contains 1,870 putative protein-coding genes. Of these, 1,288 could be assigned a Clusters of Orthologous Groups (COG) number. In addition, 39 tRNA genes and 6 rRNA operons were also identified. Based on the whole-genome sequence, 99.3% average nucleotide identity (ANI) was observed between C. fetus subsp. testudinum strain 772 and C. fetus subsp. testudinum type strain 03-427, which is higher than that between C. fetus subsp. fetus 82-40 (GenBank accession number CP000487) and C. fetus subsp. venerealis 84-112 (GenBank accession number HG004426), with 92.1% and 92.2% ANI, respectively. Further, the Kyoto Encyclopedia of Genes and Genomes (KEGG) was used for pathway analysis, which identified 370 genes related to metabolic pathways and 171 genes related to the biosynthesis of secondary metabolites. The whole-genome sequence of C. fetus subsp. testudinum strain 772 provides a better understanding of virulence, adaptation, and evolution. Further studies are warranted to investigate the pathophysiological mechanism of this pathogen.

### Accession number(s).

The complete genome sequence of C. fetus subsp. testudinum 772 has been deposited in GenBank under the accession number CP027287.
